# Ungulate Browsing Maintains Shrub Diversity in the Absence of Episodic Disturbance in Seasonally-Arid Conifer Forest

**DOI:** 10.1371/journal.pone.0086288

**Published:** 2014-01-23

**Authors:** Burak K. Pekin, Michael J. Wisdom, Bryan A. Endress, Bridgett J. Naylor, Catherine G. Parks

**Affiliations:** 1 Division of Applied Plant Ecology, Institute for Conservation Research, San Diego Zoo Global, Escondido, California, United States of America; 2 Pacific Northwest Research Station, USDA Forest Service, La Grande, Oregon, United States of America; 3 Department of Forest Ecosystems and Society, Oregon State University, Corvallis, Oregon, United States of America; University of Tartu, Estonia

## Abstract

Ungulates exert a strong influence on the composition and diversity of vegetation communities. However, little is known about how ungulate browsing pressure interacts with episodic disturbances such as fire and stand thinning. We assessed shrub responses to variable browsing pressure by cattle and elk in fuels treated (mechanical removal of fuels followed by prescribed burning) and non-fuels treated forest sites in northeastern Oregon, US. Seven treatment paddocks were established at each site; three with cattle exclusion and low, moderate and high elk browsing pressure, three with elk exclusion and low, moderate and high cattle browsing pressure, and one with both cattle and elk exclusion. The height, cover and number of stems of each shrub species were recorded at multiple plots within each paddock at the time of establishment and six years later. Changes in shrub species composition over the six year period were explored using multivariate analyses. Generalized Linear Mixed Models were used to determine the effect of browsing pressure on the change in shrub diversity and evenness. Vegetation composition in un-browsed paddocks changed more strongly and in different trajectories than in browsed paddocks at sites that were not fuels treated. In fuels treated sites, changes in composition were minimal for un-browsed paddocks. Shrub diversity and evenness decreased strongly in un-browsed paddocks relative to paddocks with low, moderate and high browsing pressure at non-fuels treated sites, but not at fuels treated sites. These results suggest that in the combined absence of fire, mechanical thinning and ungulate browsing, shrub diversity is reduced due to increased dominance by certain shrub species which are otherwise suppressed by ungulates and/or fuels removal. Accordingly, ungulate browsing, even at low intensities, can be used to suppress dominant shrub species and maintain diversity in the absence of episodic disturbance events.

## Introduction

Both livestock and wild ungulates commonly occupy high elevation pastures during the summer months in many parts of the globe. In the western United States, most ranchers bring their livestock to high elevation summer pastures that are located within public forest lands [1]. The use of these inter-montane forests by large numbers of ungulates, primarily cattle and elk, poses a significant challenge to their sustainable management [2]. A related management challenge in the seasonally dry forests of western North America is the occurrence of wildfires. In order to reduce wildfire risk and protect forest resources, prescribed burning and other fuels reduction treatments such as mechanical thinning are regularly conducted in western forests [3]. Thus, the combined effects and interactions of ungulate browsing and fuels reduction treatments are highly relevant to forest management in the western United States.

Considerable research has been conducted on the separate impacts of fires [4]–[7] and ungulates [8]–[20] on forest vegetation. Several studies have also explored the relationship between ungulate density, which is indicative of browsing pressure, and vegetation responses with regards to various site conditions such as forest type [10], site productivity [19], stand age [11], and silvicultural practices [9], [12], [18]. However, while it has been suggested that the effects of ungulates on vegetation following disturbance may differ dramatically from those during long periods without disturbance [20], studies on the combined effects of episodic disturbances and ungulate herbivory, particularly at variable intensities, in forests are lacking [2]. Consequently, little is known regarding forest vegetation responses to differing levels of ungulate browsing pressure following prescribed fire and other disturbances associated with fuels reduction treatments.

Both episodic disturbances [4]–[7] and ungulates [10]–[12], [15], [16] alter the relative abundances of different plant species and life forms through direct and indirect mechanisms. For example, frequent fires reduced shrub dominance, which allowed the abundance and number of rare species to increase in the understory of temperate eucalypt forests in Australia [4]. In deciduous forests of North America, increased browsing pressure by white-tailed deer (*Odocoileus virginianus*) led to loss of preferred woody species which in turn resulted in increased abundance of less palatable understory plants such as sedges [15]. Vegetation responses to interactions between ungulate browsing and fire are thus likely to depend on how species trajectories respond to each disturbance. For example, if both ungulates and fire reduce the cover of the same shrub species, then their effects may be additive. However, if ungulates prefer to browse on only some of the shrub species that are reduced by fire, than those that are not browsed may gain a competitive advantage and increase in abundance more greatly with the presence of ungulates following fire than under ungulate exclusion. Conversely, in absence of fire and ungulate browsing, a few shrub species may gain dominance and overcrowd or competitively exclude other species in the understory .

The response of vegetation diversity to ungulates can also vary depending on browsing frequency and intensity [17] both of which contribute to browsing pressure. In temperate forests, changes in ungulate browsing pressure often result in different trajectories of plant community composition [12], [16], and plant diversity tends to peak at an intermediate level of browsing pressure [11]. Thus, while excessive browsing may lead to loss of plant species, light to moderate browsing pressure is likely to increase diversity [17] by reducing abundance of dominant shrubs and maintaining a more even distribution of abundance across different species [13], [21], [22]. In coniferous forests of the western United States, it has been suggested that reducing the number of cattle on the landscape would generally benefit native perennial plant species [23]. However, complete ungulate exclusion has also been shown to reduce plant diversity in this ecosystem [21]. Thus, in order to guide management decisions that protect biodiversity and other forest resources in the western United States, the responses of native perennial vegetation to different levels of ungulate browsing pressure, and following different disturbance regimes, must be better understood. To this end, we assessed the role of ungulate browsing in controlling perennial vegetation dynamics in seasonally-arid conifer forests of northeastern Oregon with respect to fuels reduction treatments.

We measured the compositional and diversity responses of native forest shrubs (shrub species diversity, and evenness which is indicative of dominance) to variable browsing pressure over several years from dominant ungulate herbivores, cattle and elk, at sites where fuel loads were mechanically removed and followed by prescribed fire, and at sites with no history of fire or fuels removal for 40 years (referred to fuels treated and non-fuels treated respectively from here on). We hypothesized that vegetation responses to browsing would vary between fuels treated and non-fuels treated sites, and that the greatest level of diversity would be achieved at moderate levels of browsing pressure, particularly at non-fuels treated sites where shrub cover and dominance is high.

## Methods

### Ethics statement

Research was conducted under approval and guidance by an Institutional Animal Care and Use Committee (IACUC 92-F-0004), as required by the United States Animal Welfare Act of 1985, following the IACUC protocol for conducting cattle, elk, and mule deer research in our study area. Permission to perform the study was given by the USDA Forest Service Pacific Northwest Research Station.

### Study Area

**Figure 1:**
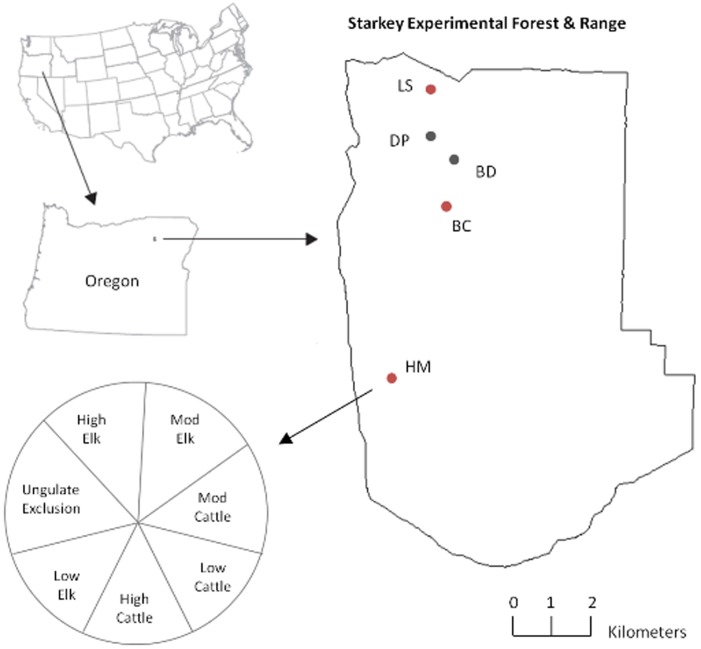
Location and design of ungulate browsing treatments at five sites at the Starkey Experimental Forest and Range in northeastern Oregon. Fuels treated sites are indicated with red dots and non-fuels treated sites in gray dots.

Our study was carried out within the Starkey Experimental Forest and Range (SEFR) in the Blue Mountains Ecological Province of northeast Oregon (Figure 1), approximately 50 km southwest of La Grande, Oregon (45°12′N, 118°3′W). The 10,000-ha SEFR has been the site of long-term research on cattle, mule deer, and elk during the past 50 years [24], and has one of the most extensive datasets on ungulate-environmental relations ever acquired [25], [26].

Conditions at SEFR are typical of montane forest types of interior western North America [24], [25]. These dry forests are the most common of all forest types in the western United States, occurring on millions of ha of public lands [24]. They are also co-occupied by high densities of domestic and wild ungulates, and thus provide an ideal setting for evaluation of browsing effects [24], [26]. Elevations at SEFR range from 1200–1500 m and most precipitation occurs as winter snow or spring rain, with a predictable drought during late summer-early fall [24]. Forest stands are generally dominated by Douglas-fir (*Pseudotsuga menziesii*) and grand fir (*Abies grandis*) [27], interspersed with grasslands and meadows [24]. Dry forests of the western United States such as those of SEFR have an inherently high level of spatial heterogeneity, owing to highly variable micro-site conditions of soil, aspect, and slope that affect the moisture available for plant growth [25].

In addition to Douglas-fir and grand fir, other canopy tree species present in these stands include ponderosa pine (*Pinus ponderosa*), western larch (*Larix occidentalis*), lodgepole pine (*Pinus contorta*), and Englemann spruce (*Picea engelmannii*). Common understory grass and grass-like species include Idaho fescue (*Festuca idahoensis*), elk sedge (*Carex geyeri*), pinegrass (*Calamagrostis rubescens*), western fescue (*Festuca occidentalis*), Kentucky bluegrass (*Poa pratensis*), and annual bromes (*Bromus spp.*). Common forbs include lupine (*Lupinus spp.*), strawberry (*Fragaria spp.*), tall annual willowherb (*Epilobium paniculatum*), and western yarrow (*Achillea millefolium*). Common shrub species include bearberry (*Arctostaphylos uva-ursi*), big huckleberry (*Vaccinium membranaceum*), grouse huckleberry (*Vaccinium scoparium*), rose (*Rosa spp*), snowberry (*Symphoricarpos albus*), shinyleaf spiraea (*Spiraea betulifolia lucida*), twinflower (*Linnaea borealis*), sticky currant (*Ribes viscosissimum*), prickly currant (*Ribes lacustre*), raspberry (*Rubus spp.*), Saskatoon serviceberry (*Amelanchier alnifolia*), black hawthorn (*Crataegus douglasii*), snowbrush (*Ceanothus velutinus*), willow (*Salix spp.*), oceanspray (*Holodiscus discolor*), wax currant (*Ribes cereum var. cereum*), orange honeysuckle (*Lonicera ciliosa*), Scouler's willow (*Salix scouleriana*), elderberry (*Sambucus spp.*), mountain ash (Sorbus scopulina), black cottonwood (*Populus balsamifera ssp. trichocarpa*), and boxleaf myrtle (*Paxistima myrsinites*).

### Fuels reduction treatments

Fuels reduction treatments were applied to grand fir and Douglas-fir stands (between 10 and 50 ha in size) in SEFR between 2000–2003 [28], [29]. Sites for fuels treatment were selected based on the high levels of fuels identified by resource managers [24]. The sites were most representative of conditions that managers deemed in need of fuels treatment to reduce the probability of undesired stand-replacement fires.

Stands were mechanically thinned with a feller-buncher to reduce fuel loadings to <35 tons/ha, compatible with fuel loads considered unlikely to carry stand-replacement fires [29], [30]. The stands were broadcast burned following mechanical thinning and removal of fuels [28]. Controlled burns were implemented during the fall of the same year, or on occasion, the following year due to time and logistical constraints. These treatments were designed and implemented in a manner typical of fuels reduction activities implemented by managers across dry forests of the western United States [3].

### Site establishment

Following fuels reduction treatment, exclosures ranging in size from five to seven hectares were established at five mixed Douglas-fir and grand-fir forest sites in the SEFR: Half Moon (HM), Louis Spring (LS), Bally Camp (BC), Doug Prairie (DP), and Bee Dee (BD) (Figure 1). Three (LS, BC, HM) of the five exclosures were constructed on fuels treated stands (Figure 1). The other two exclosures (DP, BD) were constructed where no fuels reduction treatments or any other silvicultural treatments had been implemented in >40 years. Mean annual precipitation ranged from 600 to 700 mm across the sites for the 30 year period between 1981–2010 [31]. The non-fuels treated sites were similar in forest structure and composition to pre-treatment conditions of the fuels treated sites [28].

The exclosures were established at each site by constructing a fence eight feet in height that excluded all ungulates (cattle, elk, and mule deer), but allowed for other wildlife to pass under, over, or through. The exclosures were constructed in the year following the fuels treatments. The size and shape of each exclosure varied with site conditions, including topography, slope, forest structure, and the shape of the forest patch to minimize site variation within and among exclosures. The ungulate exclosures were divided into seven, roughly equal-sized paddocks (ranging from 0.73–1.1 ha per paddock, depending on the exclosure) in which seven different levels of cattle and elk herbivory treatments were randomly assigned and implemented (Figure 1). The levels included low, moderate, or high late summer browsing by elk or by cattle and one level of total ungulate exclusion.

### Browsing trials

Browsing intensity for each treatment level was defined in terms of the number of days per ha that elk and cattle use Douglas-fir and grand fir habitat types in the interior northwestern United States, broken down into three levels of forage utilization: high—45% utilization; moderate—30% utilization; and low—15% utilization. These levels typify the range of grazing and browsing use established for cattle on summer ranges in Douglas-fir and grand fir habitat types on public lands like those at SEFR [32]. Public land grazing policies typically do not allow a level of forage utilization by cattle that exceeds the high level that we established, nor is it common that utilization levels by cattle on public lands occur below the level we established [32]. Consequently, our levels of forage utilization encompass the broad range of levels that typically occur on public grazing allotments in the western United States.

Stocking density (SD, number of days per ha) for each type of ungulate was then calculated using standard forage allocation procedures [32], based on the specified level of forage utilization (FU, expressed as a proportion), the available forage for each ungulate (AF, kg/ha), and the average daily forage intake of each ungulate (DFI, kg/ha/day) as SD = (FU*AF)/DFI. Using this formula, stocking densities were determined as follows: (1) low elk—8 days/ha; (2) moderate elk—16 days/ha; (3) high elk—32 days/ha; (4) low cattle—10 days/ha; (5) moderate cattle—20 days/ha); and (6) high cattle—30 days/ha). Use of this algorithm to establish stocking densities of domestic ungulates, and wild ungulate equivalencies, is a conventional method based on standard forage allocation procedures used on public grazing allotments throughout the western United States [32] and encompass the range of cattle and elk densities that typically occur on public ranges during summer in the western United States.

The calculated stocking densities were then refined so that the final number of cattle days per ha and elk days per ha for each treatment level corresponded to what was logistically feasible to implement with the tractable animals. Moreover, the number of animals must be compatible with each ungulate's group behavior for foraging and the need to complete the trials in as few days as possible to minimize changes in forage availability and phenology. Consequently, we used 4 elk and 6–8 cattle per trial to most efficiently approximate the calculated stocking densities. The diets of elk and cattle tend to converge during late summer in grand fir and Douglas-fir forests [33] when both ungulates select strongly for nutritious shrubs because grasses and forbs senesce and are of low quality following the onset of summer drought [34]. We thus carried out the browsing treatments each year during August for six years in a row, because we hypothesized that browsing has the greatest effect on shrub abundance and structure during this time. Moreover, our focus on shrubs was based on the limited occurrence of nutritious, deciduous shrubs in dry forests of the western United States, which has been hypothesized to be a direct result of high browsing pressure by a combination of wild and domestic ungulates during late summer and fall.

### Vegetation sampling

Permanent 40 m^2^ plots (4×10 m) were systematically established in each paddock. Due to differences in paddock size and layout, the number of plots per paddock varied (from 12–18), though the percent of each paddock sampled remains similar (7–9%). Plot locations were determined using real-time differential global positioning system placed in a geographic information system (GIS). Plots were located 15 m apart along transect lines, with each transect line spaced 15 m apart from one another within each paddock. Edges of paddocks were excluded from sampling with a 5 m buffer, and a random starting point was used to determine the starting point of each transect (ranged from 5–10 m from the outer edge of exclosure).

The absolute and relative cover of shrub species were estimated using the line-intercept method, with transects running along the center of each 4×10 m plot. Within each 4×10 m plot, all shrub stems ≥1 m in height were identified, counted, and their height measured to the nearest cm. Due to their high abundances, stems <1 m in height were identified, enumerated, and their height and cover measured in a smaller 2×10 m plot through the center of each permanent 4×10 m plot. Shrub abundances were adjusted by dividing the number of stems by the area of the sampling plot area prior to analyses. Sampling occurred in July just before implementation of the browsing treatments, and again in July, after six years of the browsing treatment. Shrub diversity and evenness were calculated for each plot using all species with Simpson index using the *diversity* function in the vegan package [35] in the statistical computing environment R [36].

The overstory has a strong influence on understory vegetation dynamics in western coniferous forests [37]. Thus, we also measured the basal area of overstory trees to include as a covariate in our modeling of understory shrub dynamics. To calculate tree basal area, we recorded the diameter at breast height (DBH) of all trees within each of the 4×10 m plots. Because of low densities of trees >20 cm diameter at breast height, we extended our plot size to 8×10 m to measure these larger trees. Tree basal areas were then adjusted for sampling plot area prior to analyses.

### Data analysis

Multivariate analyses were carried out to compare the relative changes in shrub species composition (i.e., the relative abundance of different shrub species) across un-browsed paddocks and paddocks under differing levels of browsing pressure by elk and cattle separately. A Non-metric Multidimensional Scaling was conducted using the *metaMDS* function in the R package vegan [35] with Bray Curtis distance to determine the relative compositional similarity of paddocks. Compositional trajectories were plotted for each paddock at non-fuels treated and fuels treated sites using the R package ggplot2 [38].

Because of the nested structure of our sampling design, ungulate treatment effects on vegetation structure were assessed with Generalized Linear Mixed Models (GLMM; [39]) with the *glmmPQL* function of the MASS package [40] in R. The effect of browsing by elk and cattle were tested separately on the response variables (i.e., change in shrub diversity and evenness) for paddocks at fuels treated and non-fuels treated sites. All GLMMs included a random effect defining nested plots within individual treatment paddocks. The fixed effects in models included the three levels of browsing pressure which were compared to the ungulate exclusion (no browsing) treatment. Models were initially run with both Gaussian and Poisson distributions and the distribution with the best fit was used in the final model. Total tree basal area was initially included as a covariate for all models but removed because it did not have a significant effect (P>0.1) for any of them. Figures displaying changes in shrub cover, height and diversity over the six years were made using the base *plot* function in R [36].

## Results

### Shrub cover and height across paddocks

**Figure 2:**
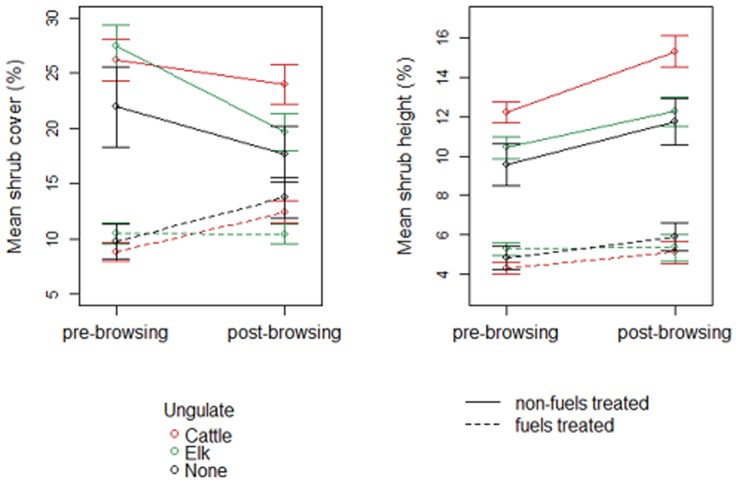
Pre and post (six years following initiation) browsing treatment initiation values for shrub cover and height of study paddocks. Mean and standard error are shown for paddocks browsed by cattle and elk, and un-browsed paddocks, at non-fuels treated and fuels treated sites seperately.

Initial mean shrub cover and height were higher at paddocks within non-fuels treated sites than paddocks within fuels treated sites (Figure 2). Mean shrub height increased slightly or remained unchanged across browsed and un-browsed paddocks within non-fuels treated and fuels treated paddocks (Figure 2). Mean shrub cover also increased slightly or remained the same within fuels treated sites (Figure 2). In contrast, at non-fuels treated sites, mean shrub cover decreased slightly but remained higher than the mean shrub cover at fuels treated sites post-treatment (Figure 2).

### Compositional responses to browsing

**Figure 3:**
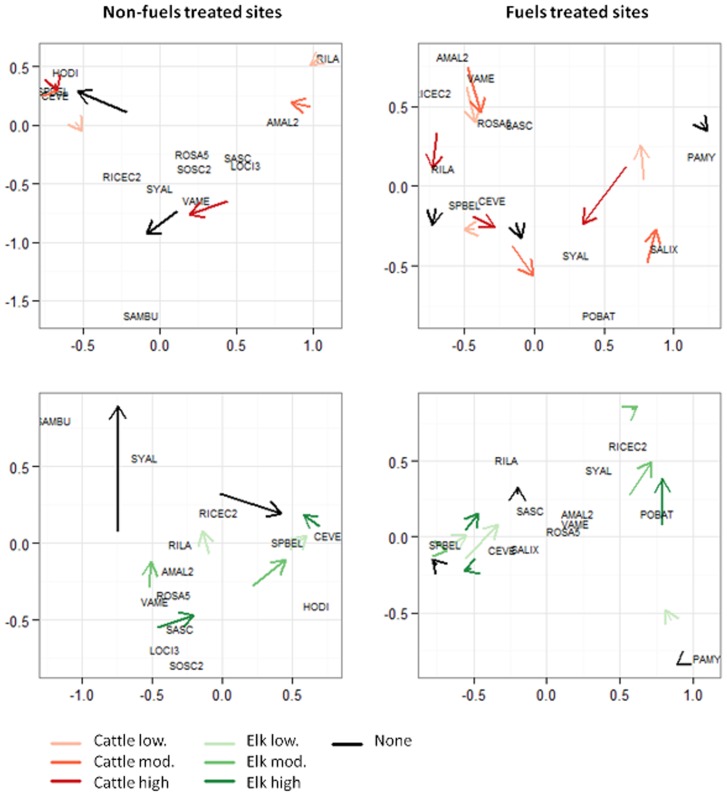
Multidimensional scaling showing change in shrub composition from the year prior to browsing initiation to six years after at fuels treated and non-fuels treated sites. The compositional trajectories of paddocks browsed by cattle and elk at three levels of browsing pressure (low, mod., high) are shown with red and green arrows indicating their pre-treatment and post-treatment composition respectively. Compositional trajectories of un-browsed paddocks (none) are shown with a black arrow. The role of individual shrub species in driving the compositional trajectories is indicated by the location of each species on the plots. Shrub species included where they occur are *Arctostaphylos uva-ursi* (ARUV), *Vaccinium membranaceum* (VAME), *Vaccinium scoparium* (VASC), *Rosa spp.* (ROSA5), *Symphoricarpos albus* (SYAL), *Spiraea betulifolia lucida* (SPBEL), *Linnaea borealis* (LIBO3), *Ribes viscosissimum* (RIVI3), *Ribes lacustre* (RILA), *Rubus spp.* (RUBUS), *Amelanchier alnifolia* (AMAL2), *Crataegus douglasii* (CRDO2), *Ceanothus velutinus* (CEVE), *Salix spp.* (SALIX), *Holodiscus discolor* (HODI), *Ribes cereum* (RICEC2), *Lonicera ciliosa* (LOCI3), *Salix scouleriana* (SASC), *Sambucus spp.* (SAMBU), *Sorbus scopulina* (SOSC), *Populus balsamifera spp. Trichocarpa* (POBAT), *and Paxistima myrsinites* (PAMY).

At non-fuels treated sites, the change in shrub composition was much greater at un-browsed paddocks compared to browsed paddocks, regardless of browsing intensity or ungulate type (Figure 3). The compositional trajectories of un-browsed paddocks were largely driven by increases in abundance of elderberry (SAMBU) or a combination of snowbrush (CEVE), shinyleaf spiraea (SPBEL), and oceanspray (HODI) (Figure 3).

In contrast, un-browsed paddocks showed minimal change in shrub composition at fuels treated sites (Figure 3). The amount and direction of compositional change was highly variable across browsed paddocks, with paddocks with high and moderate browsing pressure generally showing a greater change in composition than the rest (Figure 3).

### Diversity and evenness responses to browsing

Shrub species diversity and evenness were strongly affected by the interaction between fuels treatment and browsing at all three levels (Table 1) indicating that browsing effects on shrub diversity are not consistent between fuels treated and non-fuels treated sites. Browsing effects on diversity were thus tested separately at fuels treated and non-fuels treated sites (Table 2).

**Table 1:** Mixed model results showing intercept (*Int.*), model coefficients, estimate (*Est.*) and standard error (*SE*), and P-value (*P*) of interaction effects between fuels treatment and browsing pressure on shrub species diversity and evenness.

Ungulate	Browsing pressure		Response	*Int.*	*Est.*	*SE*	*P*
Cattle	Low	x fuels treat.	*Diversity*	1.75	0.177	0.045	0.002
			*Evenness*	2.12	0.121	0.035	0.005
	Mod.	x fuels treat.	*Diversity*	1.75	0.145	0.046	0.008
			*Evenness*	2.12	0.098	0.035	0.017
	High	x fuels treat.	*Diversity*	1.75	0.173	0.046	0.003
			*Evenness*	2.12	0.130	0.035	0.003
Elk	Low	x fuels treat.	*Diversity*	1.75	0.126	0.039	0.007
			*Evenness*	2.12	0.097	0.029	0.006
	Mod.	x fuels treat.	*Diversity*	1.75	0.165	0.039	0.001
			*Evenness*	2.12	0.117	0.029	0.002
	High	x fuels treat.	*Diversity*	1.75	0.125	0.039	0.008
			*Evenness*	2.12	0.088	0.029	0.011

Degrees of freedom is 27 for all models.

**Table 2:** Mixed model results showing effect of ungulate browsing on shrub species diversity and evenness at non-fuels treated and fuels treated sites.

Ungulate	Browsing pressure	Response	Non-fuels treated sites				Fuels treated sites			
			*Int.*	*Est.*	*SE*	*P*	*Int.*	*Est.*	*SE*	*P*
Cattle	Low	*diversity*	1.61	0.217	0.070	0.036	0.70	0.001	0.040	>0.1
		*evenness*	2.03	0.135	0.049	0.051	0.90	0.011	0.041	>0.1
	Mod.	*diversity*	1.61	0.193	0.071	0.053	0.70	0.036	0.041	>0.1
		*evenness*	2.03	0.125	0.049	0.066	0.90	0.037	0.041	>0.1
	High	*diversity*	1.61	0.205	0.070	0.043	0.70	0.016	0.040	>0.1
		*evenness*	2.03	0.146	0.049	0.041	0.90	0.007	0.042	>0.1
Elk	Low	*diversity*	1.61	0.172	0.061	0.048	0.71	0.037	0.032	>0.1
		*evenness*	2.03	0.118	0.041	0.044	0.89	0.019	0.034	>0.1
	Mod.	*diversity*	1.61	0.186	0.060	0.036	0.71	0.040	0.032	>0.1
		*evenness*	2.03	0.123	0.040	0.038	0.89	0.035	0.034	>0.1
	High	*diversity*	1.61	0.156	0.062	0.065	0.71	0.038	0.031	>0.1
		*evenness*	2.03	0.105	0.041	0.064	0.89	0.009	0.033	>0.1

**Figure 4:**
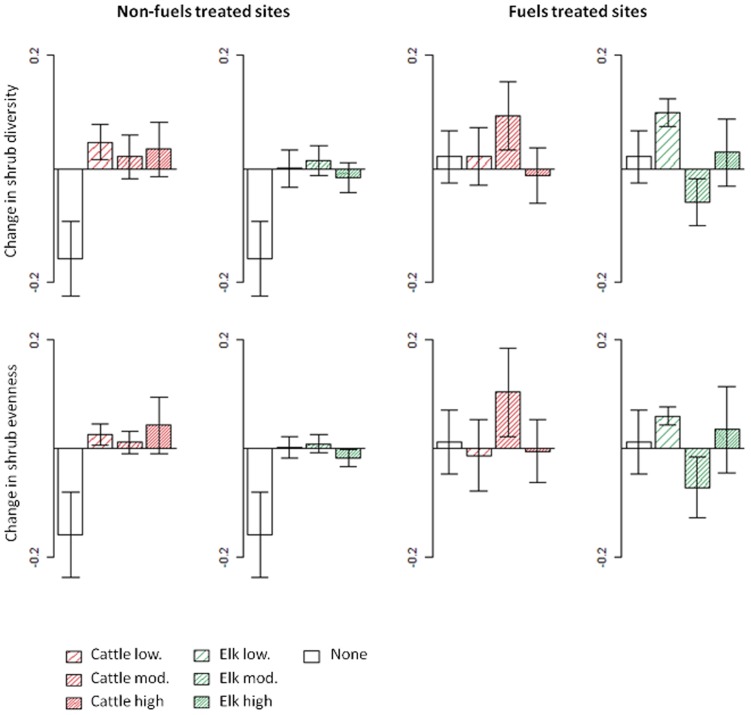
Change in shrub species diversity and evenness from the year prior to browsing initiation to six years after at non-fuels treated and fuels treated forest sites. Mean and standard error of paddocks under different levels of browsing pressure (low, mod., high) by cattle and elk is compared to mean and standard error of un-browsed paddocks (none).

The intercept (*Int.*), coefficients, estimate (*Est.*) and standard error (*SE*), and P-value (*P*) are shown for each model. The degrees of freedom is 4 for non-fuels treated sites and 8 for fuels treated sites. P-values indicate significance of difference in the mean response between each browsing pressure group from non-browsed paddocks as illustrated in Figure 4.

In non-fuels treated sites, shrub species diversity and evenness generally did not change over the study period in browsed paddocks regardless of ungulate type or browsing pressure (Figure 4). However, both species diversity and evenness were reduced substantially in paddocks where ungulates were excluded compared to paddocks browsed by elk and cattle at all three levels of browsing pressure (Figure 4; Table 2). In contrast, the changes in shrub diversity and evenness were minor and not significantly different between browsed and un-browsed paddocks at fuels treated sites (Figure 4; Table 2).

## Discussion

Our results demonstrate that ungulates have an important role in maintaining plant diversity in western forest ecosystems in the absence of episodic disturbance events such as fire and/or mechanical fuels removal. This finding agrees with Rambo and Faeth [21] who observed that plant diversity is reduced when ungulates are excluded from a Ponderosa pine – grassland ecosystem in Arizona. Episodic disturbances, whether occurring naturally or through an anthropogenic management regime, reduce dominance of large understory woody vegetation [4], [7]. This reduction in dominant woody species often provides a competitive advantage to smaller, less abundant species found in the local soil seed bank [4], [41], [42]. At non-fuels treated sites in our study, un-browsed paddocks displayed much stronger compositional changes, and lower species diversity and greater species dominance (indicated by decreasing evenness) than browsed paddocks. This suggests that in the absence of episodic disturbance and ungulate browsing, which also tends to reduce shrub dominance [11], [14], certain shrub species increase in dominance and competitively exclude other shrub species leading to reduced overall diversity.

Episodic disturbances provide new growth that is often highly preferred by ungulates [2]. Thus, it may be expected that ungulates also have a strong impact on vegetation dynamics immediately following a ground disturbance such as timber harvest, fuels removal, or fire [18], [19], [30]. However, we found that un-browsed paddocks did not display significantly different diversity responses than browsed paddocks at fuels treated sites. A likely explanation for this is that the mechanisms that reduced shrub diversity at un-browsed paddocks in non-fuels treated sites, i.e., increased shrub dominance and overcrowding, were not evident in these early succession sites [43]. Because shrub cover and height were much lower at fuels treated sites regardless of browsing treatment, it is likely to take longer than 6 years of ungulate exclusion for negative effects on shrub diversity to be evident following fire or other fuels treatment regimes involving the removal of woody vegetation in the understory in these forest stands. Several other studies have shown that the effects of ungulates on plant diversity may or may not be evident depending on pre-existing site conditions such as canopy cover, stand age, and silvicultural treatments [10]–[12], [16], [18]. These findings and those of our study demonstrate the importance of taking recent disturbance history and/or local site conditions into account when assessing ungulate effects on vegetation diversity.

While we hypothesized that diversity would be highest at moderately browsed paddocks, shrub diversity was consistently higher at all three levels of browsing pressure compared to ungulate exclusion, and no differences were observed among paddocks under different levels of browsing pressure. This suggests that relatively little browsing, 8 and 10 days per hectare annually for elk and cattle respectively, is needed to suppress dominant shrub species and maintain shrub diversity in this ecosystem. Furthermore, diversity is not reduced under higher levels of browsing pressure, of up to 32 and 30 days per hectare annually for elk and cattle respectively. It has been suggested that reduced diversity occurs once browse-tolerant species become dominant [17], which may require either more than 32 days of browsing per hectare or more than six consecutive years at this rate in our study area.

Furthermore, because we conducted our browsing treatments towards the end of the summer when the diet of elk and cattle converge, we cannot conclude from our findings that perennial understory vegetation in western conifer forest is insensitive to browsing pressure or ungulate type. Cattle and elk may have different effects on shrub dominance and diversity patterns at the beginning of the growing season, particularly with high browsing pressure. Accordingly, browsing effects at different times of the year need to be determined to assess how much browsing pressure is required to maintain diversity when herbivory occurs during the spring, early summer, late summer/fall, or a combination of the three with some browsing in each season. The latest scenario is the most realistic since elk and other wild ungulates are likely to visit these sites several times over the growing season.

Our findings have important implications for both livestock and wild ungulate management in dry forests of the western United States. First, management decisions to modify ungulate densities on the landscape will result in changes in abundance of different shrub species that could be based on a priori designs to meet vegetation management objectives for other resources, such as the desire to increase abundance of certain shrubs as habitat for wildlife. Second, while specific shrub responses may vary by type of ungulate and by the background site conditions in which herbivory occurs, either cattle or elk can be used to maintain shrub diversity in the absence of episodic disturbances. Consequently, management decisions that alter ungulate browsing patterns should take into consideration other current forest management activities and disturbances that may alter inter-specific interactions, such as prescribed burning and fuels removal. These considerations are particularly important to sustaining biodiversity in seasonally arid and fire-prone forest ecosystems of the Western United States where natural and human-induced disturbances exert strong control on vegetation.
